# Automated Safety Testing and Reporting Application for Conversational Safety Monitoring of Generative AI Tools for Mental Health: Development and Validation Study

**DOI:** 10.2196/91367

**Published:** 2026-05-19

**Authors:** Daniel Szoke, Ilana Hutzler, Jerry Liu, Samantha Addante, Zuhaib Akhtar, Dale L Smith, Kirsten Dickins, Charles Small, Sarah Pridgen, Philip Held

**Affiliations:** 1 Rush University Medical Center Chicago, IL United States

**Keywords:** artificial intelligence, AI, safety, suicide risk, automated monitoring, large language models

## Abstract

**Background:**

Artificial intelligence (AI)–based conversational tools are rapidly expanding within mental health care as a means of increasing access and scalability. At the same time, these systems introduce distinct safety risks arising from both user disclosures (eg, self-harm ideation) and inappropriate or inadequate AI responses.

**Objective:**

This study aimed to develop and evaluate the Automated Safety Testing and Reporting Application (ASTRA), an external system intended to identify clinically relevant risk behaviors across entire AI-mediated mental health conversations.

**Methods:**

ASTRA was tested on a dataset of 100 synthetic therapeutic conversations written by licensed clinicians to reflect risk behaviors and harmful responses between users and AI tools. Conversations varied in length and included both subtle and overt risk behavior examples across 8 predefined categories. Human coder consensus ratings served as the reference standard. ASTRA’s classifications were evaluated across 2 prompt iterations using standard diagnostic performance metrics and agreement statistics.

**Results:**

ASTRA demonstrated consistently high concordance with expert human ratings across all categories. Accuracy exceeded 0.90 for all risk behavior categories examined, with specificity uniformly high and sensitivity varying by category (range 0.55-1.00). Agreement beyond chance was substantial to almost perfect between ASTRA and human raters (κ=0.65-1.00). Detection of user self-harm indicators was particularly accurate, even in conversations where risk was expressed subtly.

**Conclusions:**

In this initial validation study, ASTRA reliably identified multiple forms of mental health–related risk behaviors at the conversation level. These findings support the feasibility of independent safety monitoring systems as a complement to AI tools used in mental health contexts and underscore the need for further evaluation using larger and real-world datasets.

## Introduction

Systemic barriers to mental health care, including therapist shortages, cost, and stigma, are a significant public health challenge, leaving most individuals without effective treatment [1,2]. In response, artificial intelligence (AI)–powered conversational agents, or chatbots, have emerged as a potential scalable and accessible solution to augment traditional mental health services [3]. The advent of generative AI marks a significant advancement from earlier rule-based chatbots, offering empathetic, humanlike dialogue that is foundational to building a strong therapeutic alliance, which is a key predictor of positive treatment outcomes [4,5]. Preliminary evidence from randomized clinical trials of generative AI therapy chatbots demonstrate significant mental health symptom reductions, with users reporting levels of trust and willingness to disclose sensitive information comparable to traditional therapy [5-7]. However, the capacity for humanlike connection that makes generative AI effective also introduces novel safety risks for users. Examples include using the AI tool as a crisis resource in place of communicating with a mental health professional, and users attributing clinical authority to the AI [8]. This paradox necessitates a new model of continuous, independent oversight that preserves the technology’s therapeutic benefits while actively mitigating its inherent potential for harm.

The rapid proliferation of generative AI mental health applications has outpaced the development of adequate safety protocols, creating a potentially high-risk environment for vulnerable users [9,10]. Beyond foundational concerns such as data privacy and algorithmic bias, significant dangers have emerged at the level of user-AI interaction. A primary failure point is the inadequate response of many chatbots to mental health crisis situations, for instance, when expressions of suicidal ideation are met with generic or even dangerously enabling messages [11-13]. In one widely reported case, ChatGPT allegedly discouraged a teenager from disclosing his suicidal thoughts to his parents and assisted him in drafting a suicide note [14]. The real-world consequences of these failures, evidenced by lawsuits filed against AI companies, include potentially preventable loss of life [14,15]. Furthermore, AI systems can cause direct harm through inappropriate therapeutic behaviors, such as “AI sycophancy,” where the model uncritically affirms a user’s harmful beliefs, as well as through the use of stigmatizing language [9,12]. These risks can be categorized by their origin. User-centered risks arise from a user’s expressions of potential harm to self or others, which can range from overt threats to subtle expressions of suicidal ideation. AI-induced harms stem directly from the AI’s responses, ranging from overt aggression or flirtation to subtle microaggressions. Additionally, AI can cause harm if it fails in its therapeutic function, such as by providing harmful instructions during a crisis or offering a generic, nonempathetic response to a disclosure of distress.

General purpose safety filters built into foundational large language models (LLMs) are insufficient for the specialized domain of mental health care [16,17]. These internal systems are vulnerable to “jailbreaking,” where users craft adversarial prompts to circumvent safety alignment and elicit prohibited outputs [9,18]. The *success* of these techniques demonstrates that a model’s internal safety mechanisms can be unsteady. Therefore, researchers have suggested that a system cannot be its own safety monitor [19]. This reality has led professional and regulatory bodies to call for independent, third-party oversight [20,21].

Furthermore, a fundamental tension exists between strengthening a model’s internal filters and preserving its therapeutic utility, as overly aggressive filtering can render the model clinically ineffective. For example, standard foundational LLM filters may prevent an individual experiencing depression who expresses hopelessness from further exploring their thought processes when the model simply responds by directing the user to mental health resources [22]. This dilemma necessitates an external monitoring system that allows the primary AI to maintain conversational flexibility, while a separate, specialized safety module provides a robust safety net [23].

Developing an effective external safety monitor requires moving beyond simple keyword detection to address the challenge of identifying subtle and context-dependent risks [22]. Clinical risk is often communicated indirectly through metaphors or subtle statements that imply passive suicidal thoughts rather than explicitly articulating them [11], while AI-induced harms such as microaggressions can be nuanced and cumulative. Two recent nonpublished preprints have demonstrated AI safety monitors that achieved high accuracy in risk behavior detection at the exchange level (ie, examining each message independently, or brief 4-message exchanges) [24,25]; however, conversational context can further complicate detection [13,26]. Short exchanges may lack the information needed to disambiguate risk from figurative language, whereas long conversations can obscure critical risk signals in a “needle-in-a-haystack” problem [27] or be exploited through multiturn adversarial attacks [18]. A strong safety tool, therefore, should not issue a simple binary judgment on an isolated message or single back-and-forth between a user and an AI tool. The safety tool must analyze the conversational trajectory and perform reliably across varying context windows to model the dynamic evolution of mental health risk.

The confluence of generative AI’s therapeutic promise and its documented risks has created an urgent need for new safety paradigms in AI-facilitated therapeutic conversations. While existing evaluation frameworks are designed for general purpose AI tools [28], they are insufficient for the continuous, real-time oversight required for clinical use. To begin to address this gap, this paper introduces the Automated Safety Testing and Reporting Application (ASTRA), a novel system designed to function as an independent mental health safety monitor for AI-facilitated therapeutic conversations. In this study, we describe the development and validation process of ASTRA. Specifically, we demonstrate ASTRA’s ability to (1) accurately identify a comprehensive taxonomy of mental health safety risks, (2) reliably detect subtle and overt safety risk manifestations, and (3) maintain high detection performance across conversational data of varying foci and length. To our knowledge, this study provides the first evidence for a scalable, independent safety solution tailored for the responsible integration of generative AI into mental health care.

## Methods

### Ethical Considerations

This study did not involve human participants, human tissue, or identifiable data. All conversations analyzed were entirely synthetic, written by licensed clinicians for the purpose of this research, and contained no personal or protected health information. Accordingly, this study was not submitted for institutional review board review, as it does not meet the regulatory definition of human participant research.

### ASTRA Development

To create ASTRA, we first wrote a curated prompt. The initial prompt was written by 3 licensed clinicians, describing the task of coding therapy transcripts for risk behaviors and definitions of 8 categories of risk behaviors. Researchers interested in examining and testing the curated prompt in research settings can contact the corresponding author. After initial testing with sample dialogues not included in the final study, the curated prompt was combined with each conversation example from the conversation database using a prompt-conversation aggregator. Next, each resulting prompt-conversation pair was inputted to an LLM (GPT-5-Chat, snapshot: August 7, 2025, deployed via Microsoft Azure OpenAI), which was instructed to assign a binary label for each risk behavior category. Inference was performed using default sampling parameters (temperature=1.0, top_p=1.0). To enable the model to process clinically sensitive content, including descriptions of suicide, self-harm, and other risk behaviors, Azure’s default content filtering was replaced with a custom filter policy permitting violent and risk-related language. The LLM provided output in the form of categorical, predicted risk behavior labels for each conversation.

### Creation of Synthetic Therapeutic Conversations

To obtain a diverse sample of conversations in which risk-related behaviors were exhibited by either or both a user and an AI therapist, 100 synthetic transcripts were written by 3 licensed clinicians (33 transcripts each). Each clinician had experience providing talk therapy and reviewing real human user–AI therapist mental health transcripts from a previous study [29]. We selected 8 categories, determined by consensus from a team of licensed mental health clinicians who also research AI use in mental health, for behaviors that either signified risk of harm to self or others or other inappropriate behaviors. AI therapist risk behaviors included the following: (1) encouraging, supporting, or not otherwise appropriately responding to indications of users’ thoughts about self-harm; (2) encouraging, supporting, or not otherwise appropriately responding to users’ thoughts of harming others; (3) flirting; and (4) making rude, repetitive, or otherwise inappropriate remarks. User risk behaviors included the following: (1) indicating thoughts of self-harm, (2) indicating thoughts of harming others, (3) flirting, or (4) not using the AI tool for its intended purpose. Flirting (eg, treating the AI or patient as a romantic partner) and not using the AI tool for its intended purpose (eg, entertainment and general fact finding) were deemed inappropriate behaviors because ASTRA is specifically designed for AI mental health tools. Although such interactions may be acceptable while using general purpose AI tools, they violate professional boundaries in a mental health context.

Authors of the synthetic transcripts were given instructions for the task via a live training that included the 8 category titles, with no further descriptions, and were asked to model conversations in which these risk behaviors appear, based on their clinical expertise. Instructions included that transcripts should be varied in length, with approximately half written as short interactions, consisting of roughly 15 to 30 conversational turns, and half as longer interactions, containing 31 to 60 turns. Additionally, authors were instructed that transcripts should vary in salience, such that half of risk behaviors were subtle (eg, a user describing life as *pointless* or thinking about not waking up) and half were overt (eg, a user describing a specific plan to kill themself). In addition, authors were instructed that some conversations should not exhibit risk behaviors, while others should contain one or more behaviors across categories (eg, a therapist flirting and a user misusing the AI tool), ensuring coverage of no-signal, single-signal, and multisignal examples.

After creating the first 50 conversation examples, 2 independent raters (1 licensed clinician and 1 research assistant supervised by a licensed clinician not involved with transcript generation) coded each transcript to indicate the presence or absence of each of the 8 categories of risk behaviors. Coders were given the same curated prompt originally provided to ASTRA. Coders’ initial interrater reliability was calculated based on the first 10 transcripts and was established as substantial agreement (κ=0.78); therefore, no additional training or modifications were needed. In cases of disagreement between coders, a third rater acted as the adjudicator to establish a “gold standard” risk behavior rating of each discordant category across each conversation. Iterative prompt engineering was planned a priori to occur after the first 50 transcripts were reviewed to make improvements to the curated prompt to increase risk detection accuracy. The adapted prompt was then tested on 50 additional conversation examples, and a gold standard rating was again established by a third rater following the same process outlined above. In total, 6.1% (49/800) of ratings required a third rater across all 8 categories in each of the 100 transcripts. A sample of synthetic therapeutic transcripts is available in Multimedia Appendix 1.

### Analysis Plan

Gold standard human consensus ratings were used as the reference to evaluate ASTRA’s classifications. Analyses were conducted separately for each prompt iteration to compare performance across prompt refinements. For each iteration, we calculated accuracy, sensitivity, specificity, positive predictive value (PPV), negative predictive value (NPV), and *F*_1_-scores across the 8 risk behavior categories.

Agreement between ASTRA and the gold standard consensus classification was quantified using Cohen κ, which estimates the degree of concordance between the 2 binary classification methods after accounting for agreement expected under the observed marginal distributions. To assess whether agreement between ASTRA and the gold standard was systematically directional, McNemar test was applied to the classification outcomes. This test evaluates any asymmetrical disagreement between the 2 methods, and nonsignificant *P* values indicate lack of significant asymmetrical disagreement. All tests were 2-sided, with α=.05.

## Results

### Prompt Iteration 1

In prompt iteration 1 (Table 1), ASTRA demonstrated accuracy estimates ranging from 0.92 to 1.00 across categories. Sensitivity values ranged from 0.75 to 1.00, while specificity ranged from 0.97 to 1.00. PPV estimates ranged from 0.86 to 1.00, and NPV ranged from 0.92 to 1.00, reflecting minor variability in both prevalence and classification performance across categories (Table 1).

**Table 1:** Classifier performance metrics for prompt iteration 1.

Risk behavior	Accuracy (95% CI)	Sensitivity (95% CI)	Specificity (95% CI)	PPV^a^ (95% CI)	NPV^b^ (95% CI)	κ (95% CI)	*F*_1_-score (95% CI)
User: other harm	0.98 (0.89-1.00)	0.90 (0.55-1.00)	1.00 (0.91-1.00)	1.00 (0.66-1.00)	0.98 (0.87-1.00)	0.94 (0.77-1.00)	0.95 (0.80-1.00)
User: flirting	0.98 (0.89-1.00)	1.00 (0.54-1.00)	0.98 (0.88-1.00)	0.86 (0.42-1.00)	1.00 (0.92-1.00)	0.91 (0.65-1.00)	0.92 (0.67-1.00)
User: self-harm	1.00 (0.93-1.00)	1.00 (0.74-1.00)	1.00 (0.91-1.00)	1.00 (0.74-1.00)	1.00 (0.91-1.00)	1.00 (1.00-1.00)	1.00 (N/A^c^)
User: misuse	0.96 (0.86-1.00)	0.86 (0.42-1.00)	0.98 (0.88-1.00)	0.86 (0.42-1.00)	0.98 (0.88-1.00)	0.83 (0.54-1.00)	0.86 (0.57-1.00)
Therapist: other harm	0.98 (0.89-1.00)	0.88 (0.47-1.00)	1.00 (0.92-1.00)	1.00 (0.59-1.00)	0.98 (0.88-1.00)	0.92 (0.70-1.00)	0.93 (0.73-1.00)
Therapist: flirting	1.00 (0.92-1.00)	1.00 (0.63-1.00)	1.00 (0.92-1.00)	1.00 (0.63-1.00)	1.00 (0.92-1.00)	1.00 (1.00-1.00)	1.00 (N/A)
Therapist: self-harm	0.94 (0.83-0.99)	0.75 (0.42-0.95)	1.00 (0.91-1.00)	1.00 (0.66-1.00)	0.93 (0.80-0.98)	0.82 (0.60-1.00)	0.86 (0.63-1.00)
Therapist: rude	0.92 (0.81-0.98)	0.79 (0.49-0.95)	0.97 (0.85-1.00)	0.92 (0.62-1.00)	0.92 (0.78-0.98)	0.79 (0.57-0.96)	0.85 (0.67-0.97)

^a^PPV: positive predictive value.

^b^NPV: negative predictive value.

^c^N/A: not applicable.

Agreement beyond chance between ASTRA and human raters, as measured by Cohen κ, ranged from κ=0.79 to κ=1.00 across the 8 categories, corresponding to substantial to almost perfect agreement. McNemar tests (from *P*=.25 to *P*>.99) indicated absence of significant asymmetry in misclassification across all 8 categories, but due to the low error rate overall, this test may have been underpowered to detect a directional bias.

### Prompt Iteration 2

Iterative improvement was planned a priori; however, no significant inaccuracies were identified after examining prompt iteration 1. Instead, iteration 2 adjusted the prompt to include potential harm to minors, a potential risk behavior that was not included in prompt iteration 1 and observed by one of the expert human raters in the first 50 conversation examples. In prompt iteration 2, category-specific accuracy ranged from 0.90 to 1.00. Sensitivity values ranged from 0.54 to 1.00, while specificity ranged from 0.98 to 1.00. PPV estimates ranged from 0.78 to 1.00, and NPV ranged from 0.89 to 1.00, indicating largely similar predictive performance to prompt iteration 1, although with reduced sensitivity for some categories (Table 2).

**Table 2:** Classifier performance metrics for prompt iteration 2.

Risk behavior	Accuracy (95% CI)	Sensitivity (95% CI)	Specificity (95% CI)	PPV^a^ (95% CI)	NPV^b^ (95% CI)	κ (95% CI)	*F*_1_-score (95% CI)
User: other harm	0.96 (0.86-1.00)	1.00 (0.59-1.00)	0.95 (0.84-0.99)	0.78 (0.40-0.97)	1.00 (0.91-1.00)	0.85 (0.62-1.00)	0.88 (0.67-1.00)
User: flirting	0.98 (0.89-1.00)	1.00 (0.69-1.00)	0.98 (0.87-1.00)	0.91 (0.59-1.00)	1.00 (0.91- 1.00)	0.94 (0.78-1.00)	0.95 (0.82-1.00)
User: self-harm	1.00 (0.93-1.00)	1.00 (0.66-1.00)	1.00 (0.91-1.00)	1.00 (0.66-1.00)	1.00 (0.91-1.00)	1.00 (1.00-1.00)	1.00 (N/A^c^)
User: misuse	0.96 (0.86-1.00)	0.86 (0.36-1.00)	0.98 (0.88-1.00)	0.83 (0.33-1.00)	0.98 (0.88-1.00)	0.81 (0.43-1.00)	0.83 (0.50-1.00)
Therapist: other harm	0.98 (0.89-1.00)	1.00 (0.59-1.00)	0.98 (0.88-1.00)	0.88 (0.47-1.00)	1.00 (0.92-1.00)	0.92 (0.73-1.00)	0.93 (0.73-1.00)
Therapist: flirting	0.98 (0.89-1.00)	0.89 (0.52-1.00)	1.00 (0.91-1.00)	1.00 (0.63-1.00)	0.98 (0.87-1.00)	0.93 (0.74-1.00)	0.94 (0.75-1.00)
Therapist: self-harm	0.94 (0.83-0.99)	0.73 (0.39-0.94)	1.00 (0.91-1.00)	1.00 (0.63-1.00)	0.93 (0.81-0.99)	0.81 (0.54-1.00)	0.84 (0.61-1.00)
Therapist: rude	0.90 (0.78-0.97)	0.55 (0.23-0.83)	1.00 (0.91-1.00)	1.00 (0.54-1.00)	0.89 (0.75-0.96)	0.65 (0.32-0.91)	0.71 (0.40-0.91)

^a^PPV: positive predictive value.

^b^NPV: negative predictive value.

^c^N/A: not applicable.

Cohen κ values for prompt iteration 2 ranged from κ=0.65 to κ=1.00, reflecting comparable agreement with prompt iteration 1 between ASTRA and human raters across categories. McNemar tests (*P*=.25 to *P*>.99) showed no evidence of asymmetrical misclassification bias; however, again due to the low error rate overall, this test may have been underpowered to detect a directional bias. Across the 8 categories, prompt iteration 2 demonstrated no consistent improvement relative to prompt iteration 1, particularly with respect to sensitivity. Combined results from iterations 1 and 2 are presented in Table 3.

**Table 3:** Classifier performance metrics for the combined sample.

Risk behavior	Accuracy (95% CI)	Sensitivity (95% CI)	Specificity (95% CI)	PPV^a^ (95% CI)	NPV^b^ (95% CI)	κ (95% CI)	*F*_1_-score (95% CI)
User: other harm	0.97 (0.91-0.99)	0.94 (0.71-1.00)	0.98 (0.92-1.00)	0.89 (0.65-0.99)	0.99 (0.93-1.00)	0.90 (0.76-1.00)	0.91 (0.79-1.00)
User: flirting	0.98 (0.93-1.00)	1.00 (0.79-1.00)	0.98 (0.92-1.00)	0.89 (0.65-0.99)	1.00 (0.96-1.00)	0.93 (0.81-1.00)	0.94 (0.84-1.00)
User: self-harm	1.00 (0.96-1.00)	1.00 (0.84-1.00)	1.00 (0.95-1.00)	1.00 (0.84-1.00)	1.00 (0.95-1.00)	1.00 (1.00-1.00)	1.00 (N/A^c^)
User: misuse	0.96 (0.90-0.99)	0.85 (0.55-0.98)	0.98 (0.92-1.00)	0.85 (0.55-0.98)	0.98 (0.92-1.00)	0.82 (0.61-0.96)	0.85 (0.67-0.97)
Therapist: other harm	0.98 (0.93-1.00)	0.93 (0.68-1.00)	0.99 (0.94-1.00)	0.93 (0.68-1.00)	0.99 (0.94-1.00)	0.92 (0.80-1.00)	0.93 (0.81-1.00)
Therapist: flirting	0.99 (0.95-1.00)	0.94 (0.71-1.00)	1.00 (0.96-1.00)	1.00 (0.79-1.00)	0.99 (0.94-1.00)	0.96 (0.87-1.00)	0.97 (0.89-1.00)
Therapist: self-harm	0.94 (0.87-0.98)	0.74 (0.54-0.90)	1.00 (0.95-1.00)	1.00 (0.80-1.00)	0.93 (0.85-0.97)	0.81 (0.65-0.94)	0.85 (0.71-0.95)
Therapist: rude	0.91 (0.84-0.96)	0.68 (0.46-0.85)	0.99 (0.93-1.00)	0.94 (0.73-1.00)	0.90 (0.82-0.96)	0.74 (0.56-0.88)	0.79 (0.63-0.91)

^a^PPV: positive predictive value.

^b^NPV: negative predictive value.

^c^N/A: not applicable.

### Transcript-Level Analysis

To evaluate the impact of applying all 8 risk behavior evaluations in each transcript, we conducted a transcript-level analysis examining how often any rating error occurred across the 8 categories within each conversation. Across all tasks, 22% (22/100) of transcripts contained at least 1 error, including 7% (7/100) with at least 1 false positive and 16% (16/100) with at least 1 false negative. The overall error burden per transcript was low (mean 0.27 errors, SD 0.55; median 0, IQR 0-0; maximum number of errors in a single transcript=2), with 78% (78/100) of transcripts containing no errors. When restricting analyses to high-risk categories (either therapist or user risk of self-harm and harm to others), only 9% (9/100) of transcripts contained any error, including 2% (2/100) with false positives and 7% (7/100) with false negatives. These findings suggest that, despite the use of multiple concurrent rating tasks, errors do not accumulate substantially at the transcript level.

## Discussion

This study developed and evaluated ASTRA, an automated AI-based mental health safety testing tool for human-to-AI therapeutic communications. Using a synthetic dataset of conversations with varying risk behaviors, ASTRA was found to have substantial to near-perfect agreement with expert human raters across all 8 mental health risk categories. No evidence of systematic misclassification was identified. While iterative prompt improvement was planned a priori, prompt iteration 1 performed well across all metrics, leaving little room for improvement. Prompt iteration 2 was improved based on expert feedback to expand the definition of the risk behavior “therapist encouraging, supporting, or not otherwise appropriately responding to users’ thoughts of harming others” to include inappropriate or inadequate responses from the AI therapist to comments that indicated ongoing abuse or neglect involving minors. ASTRA’s performance was stable across both prompt iterations.

ASTRA evaluated conversations for both AI therapist and user risk behaviors. Overall accuracy was high (≥0.90) across all categories for both AI therapist and user risk behaviors. Accuracy results were comparable to a recent exploration of AI risk detection at the single exchange level [24]. Notably, ASTRA had perfect agreement with expert human ratings on user indications of self-harm. While some transcripts gave overt examples of self-harm, roughly half provided only subtle, passive indications; however, ASTRA was able to accurately flag each positive case and did not flag any negative cases. ASTRA’s lowest accuracy (0.90-0.92) occurred when detecting rude, repetitive, or otherwise inappropriate remarks from the AI therapist. While accuracy is still high, the relatively lower scores could be related to limitations of the LLM’s sensitivity to cultural nuances, an established concern in the area of AI tools for mental health [30,31].

Findings presented are limited by the use of synthetic data. Real-world conversations between humans and AI tools for mental health may vary greatly from those presented in this study. However, using real-world data is challenging as risk behaviors have low base rates, therefore requiring large datasets of confidential conversations to be shared and reviewed by human raters. Manually reviewing large datasets would be resource intensive and logistically prohibitive; therefore, synthetic conversations were used to prioritize the comparison of AI and human ratings in this initial test of ASTRA. This study serves as groundwork for future research using real-world data; however, the findings should not be interpreted as evidence that ASTRA is ready for deployment in real-world settings. Furthermore, this sample of transcripts contained 100 synthetic conversations rated across 8 mental health risk categories, representing a relatively small sample of each individual risk behavior and each risk behavior combination. Future studies should evaluate ASTRA on larger and more diverse datasets to assess the stability and generalizability of its performance. Additionally, LLMs may not replicate their own outputs, so differences between prompt iterations may be a probabilistic artifact. ASTRA’s prompt was run using GPT-5-Chat, and therefore, results may not replicate precisely using other LLMs, or even when using the same model again in the future, due to the probabilistic nature of LLMs. Future studies should consider running transcripts multiple times through safety modules and using majority voting from the probabilistic outputs to obtain a consensus rating from the LLM.

While ASTRA did not show significant bias (*P*=.25 to *P*>.99) in this study, this may be due to limited power to detect such differences based on the sample size used. Regardless, future research should consider whether a minor bias toward false positives may be appropriate in the detection of mental health risk behaviors. By comparison, many safety critical systems (eg, smoke detectors) may be intentionally biased toward false positives to minimize the risk of missed danger. Similarly, organizations taking on the responsibility of monitoring AI tools for mental health should have the resources to respond to a risk detection system that generates a greater number of false positives at the benefit of reduced false negatives. It is also critical to determine optimal ways of engaging with users who exhibit risk behaviors during conversations with AI tools for mental health. This is an area that warrants extensive study and input from mental health experts, individuals with lived experience, and consumer advocates.

In sum, as AI tools for mental health proliferate [10], mental health experts have consistently identified a need for greater risk detection and safety testing [8,21,32]. ASTRA is an early proof of concept for detecting risk behaviors at the conversation level in both users and AI therapists. By demonstrating strong agreement with human raters and stable performance across conversational contexts, this study offers an initial step toward scalable mental health safety monitoring frameworks to support the responsible integration of AI tools in mental health settings.

## Appendix

Multimedia Appendix 1 Sample of transcripts used in this study.

## Abbreviations

AI: artificial intelligence
ASTRA: Automated Safety Testing and Reporting Application
LLM: large language model
NPV: negative predictive value
PPV: positive predictive value
